# NMR WaterLOGSY Reveals Weak Binding of Bisphenol A with Amyloid Fibers of a Conserved 11 Residue Peptide from Androgen Receptor

**DOI:** 10.1371/journal.pone.0161948

**Published:** 2016-09-01

**Authors:** Julia Asencio-Hernández, Bruno Kieffer, Marc-André Delsuc

**Affiliations:** Institut de Génétique et de Biologie Moléculaire et Cellulaire (IGBMC), INSERM U596, CNRS UMR 7104, Université de Strasbourg, Illkirch-Graffenstaden, France; Francis Crick Institute, UNITED KINGDOM

## Abstract

There is growing evidence that bisphenol A (BPA), a molecule largely released in the environment, has detrimental effects on ecosystems and on human health. It acts as an endocrine disruptor targeting steroid hormone receptors, such as the estrogen receptor (ER), estrogen-related receptor (ERR) and androgen receptor (AR). BPA-derived molecules have recently been shown to interact with the AR N-terminal domain (AR-NTD), which is known to be largely intrinsically disordered. This N-terminal domain contains an 11 residue conserved domain that forms amyloid fibers upon oxidative dimerisation through its strictly conserved Cys240 residue. We investigate here the interaction of BPA, and other potential endocrine disruptors, with AR-NTD amyloid fibers using the WaterLOGSY NMR experiment. We observed a selective binding of these compounds to the amyloid fibers formed by the AR-NTD conserved region and glutamine homopolymers. This observation suggests that the high potency of endocrine disruptors may result, in part, from their ability to bind amyloid forms of nuclear receptors in addition to their cognate binding sites. This property may be exploited to design future therapeutic strategies targeting AR related diseases such as the spinal bulbar muscular atrophy or prostate cancer. The ability of NMR WaterLOGSY experiments to detect weak interactions between small ligands and amyloid fibers may prove to be of particular interest for identifying promising hit molecules.

## Introduction

Bisphenol A (BPA) is a chemical additive that was widely used in the plastics industry, until growing evidence of its detrimental effect on human health was revealed. Long-term exposure to low-doses of BPA is responsible for developmental defects of the reproductive system, as well as metabolic and neurologic disorders. Higher doses of BPA display oxidative toxicity and carcinogenesis effects [1]. Multiple mechanisms have been proposed to explain the toxicity of BPA, the most important one being its ability to interfere with steroid hormone regulatory systems. Direct interactions between BPA and several steroid hormone receptors, such as the estrogen receptor (ER), estrogen-related receptor (ERR) and androgen receptor (AR) have been reported [2]. Steroid hormone receptors belong to the large family of nuclear hormone receptors defined by a common organization of three distinct domains including the ligand binding domain (LBD), the DNA binding domain (DBD) and the N-terminal domain (NTD). While the DBD and the LBD present a highly conserved three dimensional structure, the NTD of these nuclear receptors is largely intrinsically disordered and highly variable in length and composition [3]. The LBD is responsible for the ligand dependent activation of transcription, while the NTD harbours a ligand-independent activation function (AF-1) distinct from the ligand-dependent activation function (AF-2) located in the LBD. Direct evidence of BPA binding to the LBD of ER and AR has recently been provided [4, 5].

**Fig 1:**
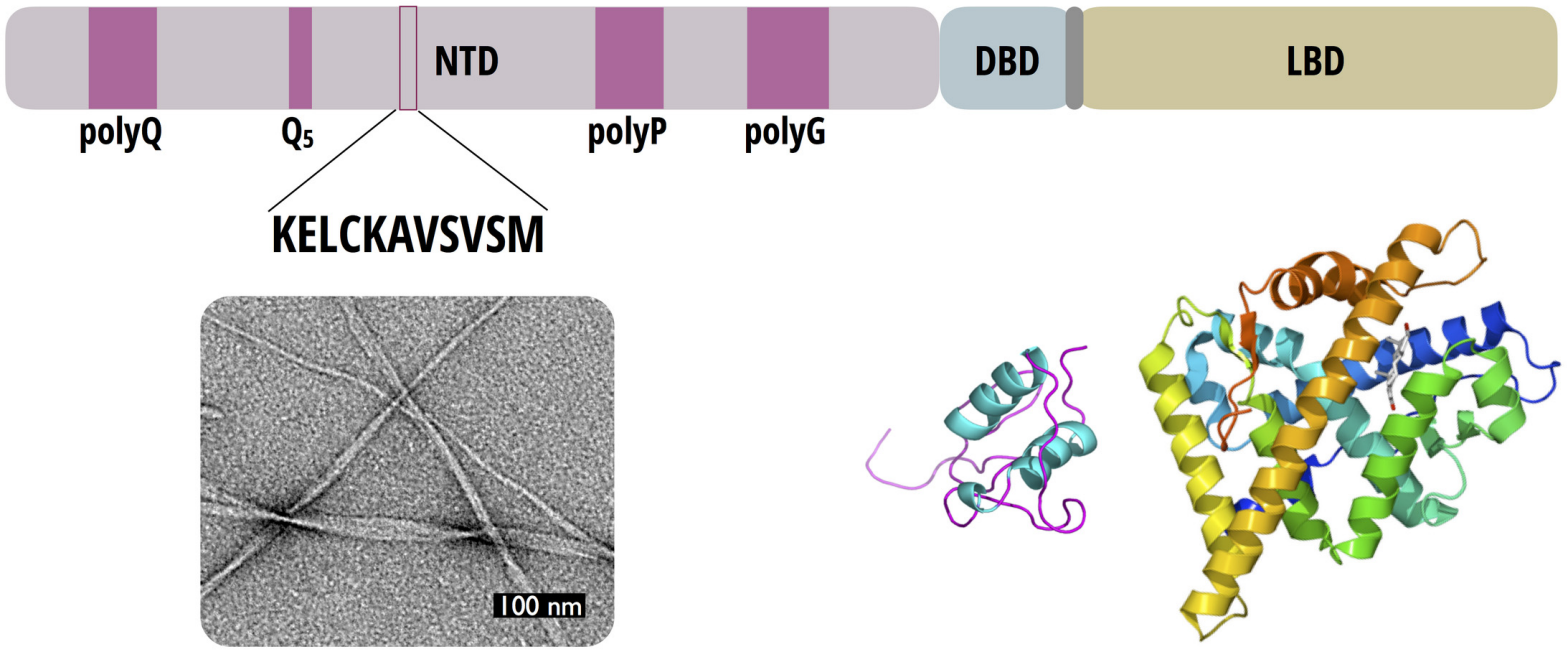
Domain organization of Androgen Nuclear Receptor. Schematic representation of AR showing the structured DBD and LBD domains, connected by the hinge region in grey and the N-terminal disordered domain (NTD). A zoom of the **P11** region shows the sequence of the peptide investigated in this work and the amyloid fibers formed upon Cys240 oxidation.

Due to their intrinsic disorder, molecular mechanisms underlying the activation function of nuclear receptor NTDs remain largely unknown. However, these domains appear as promising target sites for the development of next generation drugs able to specifically interact with the disordered domains of nuclear receptors. In the case of AR, the molecule EPI–001 and its derivatives bind to the AR-NTD and have been presented as potential drugs that promote the regression of prostate cancer, a disease that is essentially under the regulation of the androgen receptor (AR) [6–8]. A distinct feature of the AR-NTD is its large size (555 amino acids), which accounts for 66% of the whole AR sequence, and its composition that includes homopolymer stretches of glutamine (polyQ), glycine (polyG) or proline (polyP) residues (Fig 1). The polyQ sequence, composed usually of 20 to 40 residues, has been shown to be critical for the supramolecular organization of AR. The physiological properties and toxicity of the polyQ sequence depend on the number *N* of glutamines in the sequence. All variants can form amyloids [9]. Genetic polyQ expansions with *N* > 40, however, induce toxic aggregates that lead to mild androgen insensitivity and neurotoxicity, and are involved in the development of the spinal and bulbar muscular atrophy (SBMA) neurodegenerative disease [10–12]. Amyloid aggregates are still formed by the variants of AR with shorter polyQ stretches (1 ≤ *N* < 20). The proteins which include these short stretches are not directly toxic, but lead to an increased risk of prostate cancer and possibly modulate the aggressiveness of this disease [11].

The central part of the AR-NTD contains a conserved sequence of eleven residues (K237-M247) encompassing the strictly conserved Cys240. Hereafter, we refer to this 11 residue sequence as **P11**. We have recently shown that the oxidation of Cys240 promoted the formation of peptide amyloid fibers, a process that can be reversed by the addition of a reducing agent such as tris(2-carboxyethyl)phosphine (TCEP) [13].

Converging evidence suggests that AR aggregation is involved in some aspects of AR function and AR-related pathologies in a manner that is not well understood. Here, we explore possible interactions between BPA, together with several other endocrine disruptors, and the amyloid fibers formed by the **P11** motif using WaterLOGSY (WL) NMR experiments [14, 15]. We observed a weak but selective binding of these compounds to the **P11** peptide organized in amyloid fibers suggesting that BPA and related molecules may be used to target this specific form of AR.

## Materials and Methods

### Peptides

Peptide synthesis was performed on-site using a 433A Peptide Synthesizer (ABI) and Fmoc chemistry. Crude peptides were purified by reverse phase HPLC on a 250 × 21.2 mm Luna C18 100 Å, 5 *μ*m column from Phenomenex, and controlled by mass spectrometry (ESI/TOF micrOTOF II Bruker). The peptide amino acid sequences involved in this study are:

**P11** KELCKAVSVSM
**mut** KELSKAVSVSM

The peptides were desalted using a GF Superdex 10/300 column in an AKTA purifier system; the trifluoroacetic acid (TFA) was removed by performing three cycles of dilution in HCl and lyophilization as described in [16]. Accurate peptide quantifications were performed by NMR using tryptophan as internal concentration standard as described in Kæhler et al. [17]

The peptides were stored as lyophilized powder at -20°C. The concentration of the soluble peptide forms after aggregation was measured by integrating the NMR signals of the methyl region ([0.8ppm–1.0ppm]).

### Sample preparation

Stock solutions of the ligand molecules were stored in DMSO-d_6_ at 100 mM. All NMR experiments were performed in 3 mm tubes using 180 *μ*l of H_2_O containing 10% DMSO-d_6_ and 10% D_2_O v/v at final pH = 7.0. The final concentrations of the molecules were set to 1 mM for both the peptide and the ligand.

Fibers were prepared as described in Asencio-Hernández et al [13] from 1 mM peptide solution with 10% DMSO-d_6_ and 10% D_2_O incubated for 24 h at 37°C. Then, the fiber suspension was diluted to different extents (1/2, 1/5, 1/10, 1/20, 1/50 and 1/100) maintaining the concentration of both DMSO-d_6_ and D_2_O to 10% v/v.

### NMR analysis

All NMR studies were performed at 25°C on a Bruker Avance III 700 MHz spectrometer equipped with a Z-gradient cryoprobe. The spectra were processed and analyzed using NMR-notebook^®^ (NMRTEC, Illkirch, France).

1D ^1^H spectra were recorded using a 1 s presaturation pulse with a B_1_ power of 200 Hz.

WaterLOGSY experiments (WL) were performed using the ePHOGSY experiment from Dalvit et al. 2001 [15]. Selective water excitation was achieved using a 20 ms truncated Gaussian pulse with a maximum B_1_ power of 60 Hz. The mixing time was set to 1.2 s and the relaxation time was set to 6 s. (see Figs A to E in S1 Spectra).

Experiments were acquired with 256 scans for a total duration of 36 min. For all experiments, the 90^°^ pulse was between 9 and 11 *μ*s.

WL experiments in the absence of **P11** were performed for all screened molecules. All control experiments displayed a negative WL signal for the ligand resonances (see Figs H to M in S1 Spectra).

The uncertainty on the WL signal intensity values was calculated using as reference, the DMSO signal from eight different experiments.

## Results

### BPA binding to P11 peptide fibers

We first aimed at investigating BPA affinity for monomeric AR **P11** peptide using NMR, a method sensitive to molecular interactions over a broad range of affinity.

BPA requires DMSO as cosolvent to obtain aqueous solubility, but DMSO also promotes the formation of **P11** fibers. Therefore, we were unable to directly measure the affinity of BPA for the monomeric peptide. However, the addition of BPA to a solution of the **mut** peptide, where the cysteine is replaced by a serine, leaves the proton spectrum of the peptide unchanged, indicating that BPA has no measurable affinity for the mutated peptide sequence.

**Fig 2:**
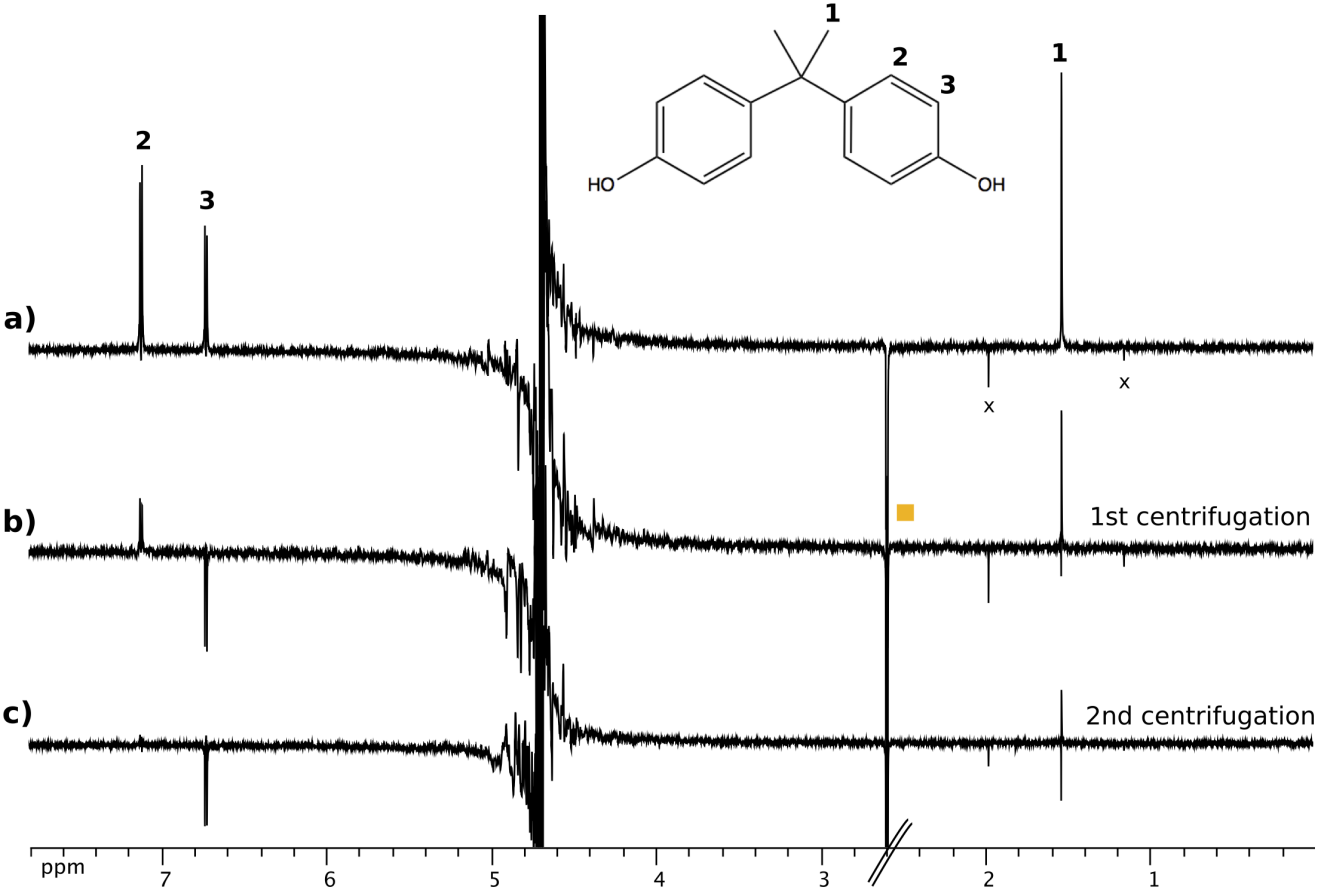
WaterLOGSY study of BPA with the P11 amyloid fibers. (a) WL difference spectrum of a 1 mM solution of BPA in presence of 200 *μ*M of **P11** peptide and 10% DMSO-d_6_. BPA peaks are labelled, the orange square indicates the methyl resonance of DMSO, and the × indicate two impurities found in the solution, possibly acetonitrile and ter-butanol. b,c) WL difference spectrum of the BPA solution featuring the same composition as in (a) after a first centrifugation step (b) and after a second one (c). Centrifugation was performed at 10000 r.p.m. for 40 min on each occasion. The mixed sign observed on the sharp methyl **1** signal apparently arises from incomplete signal cancelation during acquisition caused by instrument instabilities.

We then tested whether BPA binds to the **P11** peptide embedded in amyloid fibers. The formation of **P11** fibers involves the oxidation of the cysteine and disulfide bridge formation due to the oxidizing potential of DMSO [18] as an initial step, followed by the self-association of covalent peptide homodimers [13]. The later process leads to a progressive loss of proton NMR signals due to the peptide. During this process, the intensity of the proton resonances corresponding to the BPA were not affected, indicating the lack of strong interaction between BPA and the amyloid fibers. We then investigated the presence of weak transient interactions using WaterLOGSY (WL) technique [15] (Fig 2). This experiment is based on the detection of a transfer of magnetization from the solvent to small solute molecules, the sign of which is inverted when the solute molecules can bind to slowly tumbling macromolecules within the sample. WL is suited for fast *k*_off_ values, where *k*_off_ > 1/*T*_1_, meaning that it is adapted for the detection of weakly binding ligands [19], nevertheless this method presents a problem when working with poorly soluble molecules that lead to create artifacts and false positive responses [20]. For this reason, all molecules presented in this work were tested alone in solution and the WL response obtained was negative or null (see Figs H to M in S1 Spectra).

The presence of large positive signals for proton resonances of the BPA molecule in the WL spectrum indicates that water magnetization was transferred to BPA while bound to a large molecular object. The residual protonated DMSO peak displays a negative intensity, indicative of a direct magnetization transfer between water and DMSO only, providing us with a negative control (Fig 2).

In order to assess whether the **P11** fibrils were responsible for the magnetization transfer from water to BPA, we performed the same measurement after two steps of sample centrifugation by sedimentation to remove high molecular weight species from the solution (Fig 2). The first centrifugation step led to a significant decrease of BPA signal while the second step led to a near complete disappearance of the WaterLOGSY effect for BPA with the proton **3** resonance appearing negative and the others close to zero. No significant change of intensity was observed for the DMSO signal. These results indicate that BPA binds to the **P11** peptide only when this motif is highly ordered within fibrillar structures.

### Concentration dependence of BPA-fiber interaction

**Fig 3:**
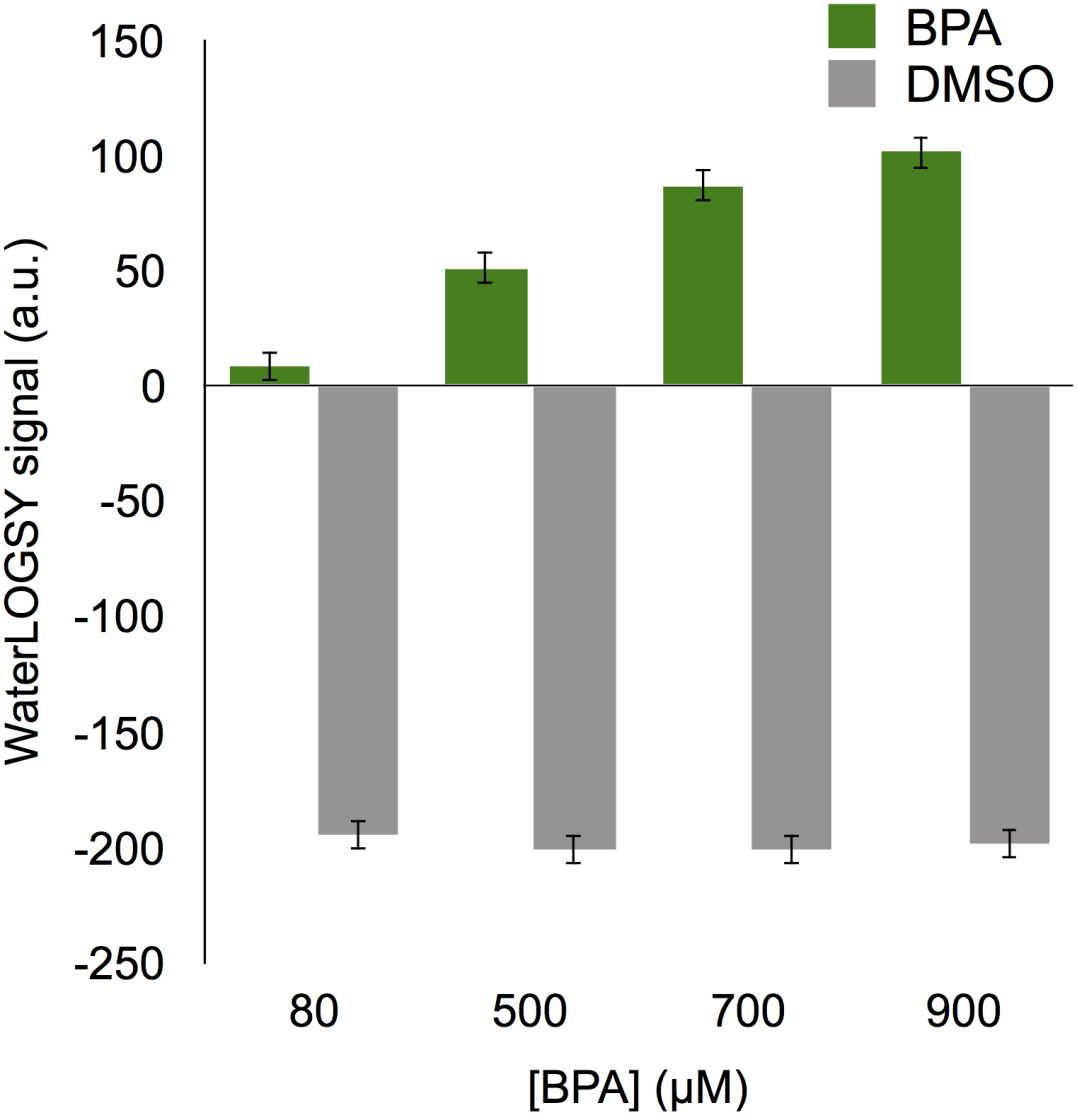
BPA concentration dependance of WaterLOGSY signal. Signal intensities from the methyl groups of BPA (in green) and the DMSO (in grey) are shown as a function of BPA concentration. The **P11** peptide concentration is 200 *μ*M.

**Fig 4:**
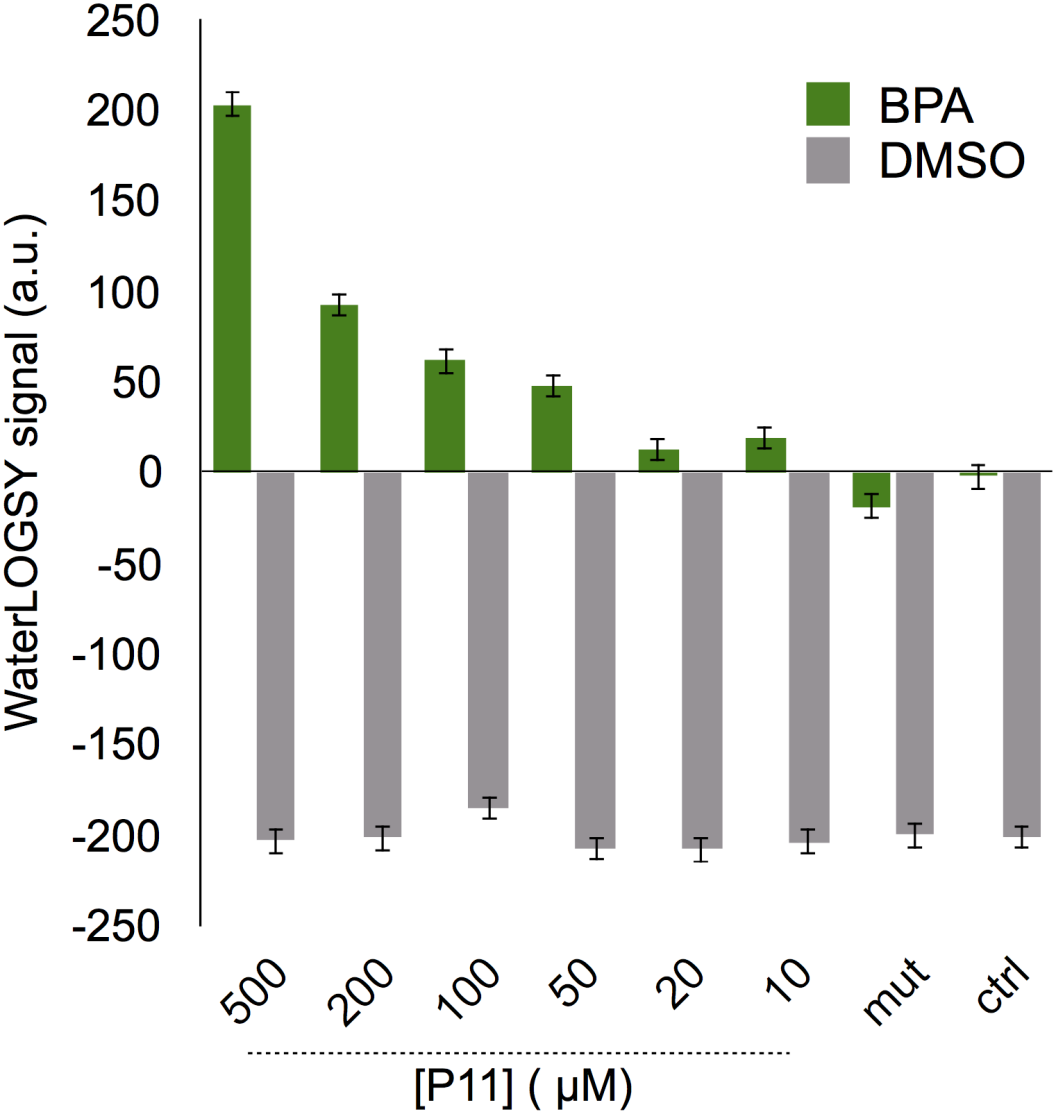
Fiber concentration dependance of WaterLOGSY signal. Signal intensities from the methyl groups of BPA (in green) and the DMSO (in grey) are shown as a function of the **P11** peptide concentration. WL signal intensities are also shown for the ligand either with 1 mM of the **mut** peptide or without fibers (ctrl), showing negative response within the experimental error.

In the WaterLOGSY experiment, several mechanisms may contribute to the signal characterizing the bound form of the ligand, rendering the extraction of quantitative information such as a binding constant difficult in the absence of severe assumptions. In the case of the interaction between a fibril and a ligand, the number of binding sites clearly presents an issue. We nevertheless characterized dose-dependent effects on WL signals to get some insight into the stoichiometry of the interaction. This experiment was performed either by measuring the signal for increasing concentrations of BPA whilst keeping the fibril concentration constant (Fig 3), or by decreasing the concentration of fibrils while keeping the concentration of BPA constant (Fig 4). Increasing the BPA concentration in a solution containing 200 *μ*M of fibrillar **P11** peptide led to a linear increase of the WL signal. The absence of saturation effect is consistent with a dissociation constant in the mM range.

A similar linear WL signal response as a function of the peptide concentration is observed when the experiment is performed with progressive dilution of **P11** peptide fibers. It is remarkable that a significant WL signal is still observed for peptide concentrations as low as 10 *μ*M. This observation may be due to the slow correlation time of the fibers which leads to high efficiency of the magnetization transfer between water molecules and bound BPA. This observation, together with the absence of saturation upon progressive addition of BPA to the peptide, suggests that numerous binding sites are likely available for BPA on the surface of the **P11** fibers.

### Specificity of BPA interaction to P11 fibers

The interaction between BPA and **P11** peptide fibers may result either from binding pockets on the surface of the fibers defined by the **P11** side chains (and therefore a function of the peptide amino acid sequence) or, less specifically, from a particular geometric organization of the peptide backbone, arguably within the fiber. In order to distinguish between these two hypotheses, we tested whether WL signal can be observed using fibers formed by homopolymers of glutamines (polyQ). We used fibers formed by Q41 peptides whose amyloid forming property is responsible for spinal and bulbar muscular atrophy [11] and Huntington’s disease [21, 22].

**Fig 5:**
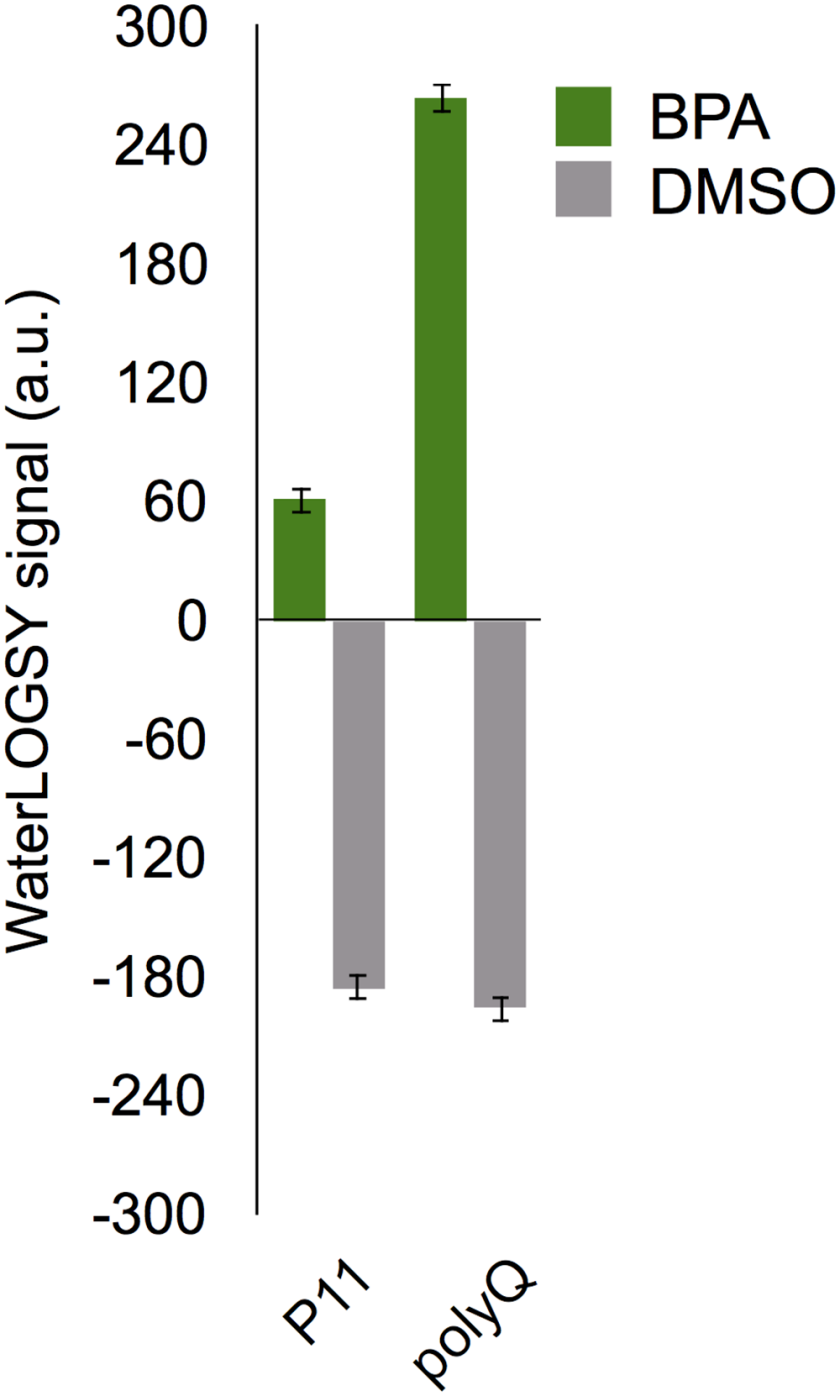
Comparison of BPA WaterLOGSY signal intensities for P11 and polyQ fibers. The WL signal intensity was measured using the methyl groups of BPA (in green) and DMSO (in grey). The concentration of **P11** and polyQ peptides was 200 *μ*M and BPA concentration is 1 mM.

The comparison of WL signal obtained from experiments performed with peptides and BPA concentrations similar to those used for the **P11** fibers is shown in Fig 5. As observed for **P11** fibers, we measured a strong positive WL signal, showing that both fibers promote strong ligand-water magnetization transfer through the weak ligand-fiber interaction. This is an indication that the recognition is mediated by molecular features that are specific to the amyloid state but independent of the peptide primary structure.

We then investigated whether other molecules known to interact with AR or with other intrinsically disordered domains display some affinity for **P11** peptide fibers using WL experiments. EPI–001 is a non-steroidal antagonist of AR derived from BPA, which binds covalently to the AR-NTD [23]. EPI–001 shares a common core chemical structure with BPA. Testosterone is the natural ligand of AR, while resveratrol and epigallocatechin gallate (EGCG) are two polyphenol compounds known for their beneficial effects on human health, and for which interactions with amyloid fibers have been suggested [24–26]. Chloramphenicol is an antibiotic whose chemical structure contains an aromatic ring that targets the bacterial ribosome. This molecule was chosen as a negative control.

**Fig 6:**
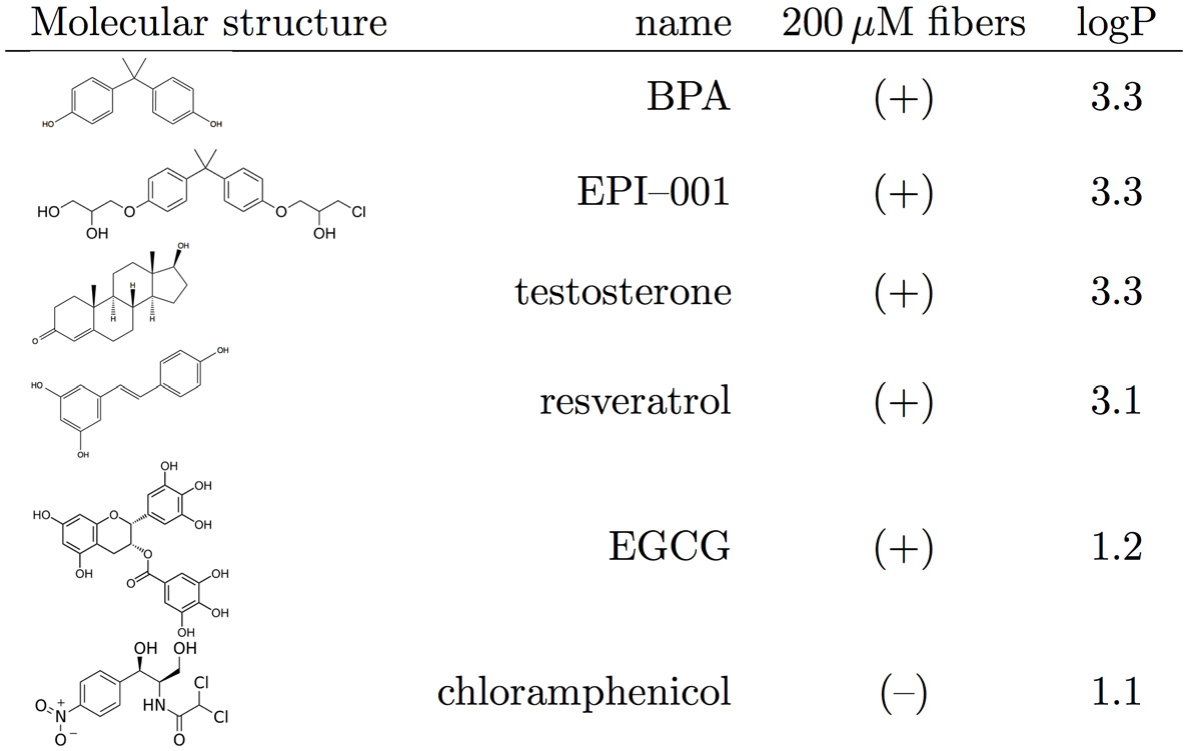
WaterLOGSY responses obtained for different molecules in presence of P11 amyloid fibers. (+) denotes a positive WL response while (–) denotes a negative signal characterizing the absence of interaction. *(Details presented in* S1 Spectra).

Stock solutions of these compounds were prepared in DMSO-d_6_ and their interactions with **P11** peptide fibers were tested with WL experiments performed at concentrations of 1 mM for the ligand and 200 *μ*M for **P11**. The results are summarized in Fig 6.

Remarkably, all the tested compounds present a positive WL signal, indicative of a weak interaction with the fibers, with the exception of chloramphenicol. This observation suggests that amyloid fibers have the ability to bind a wide range of ligands presenting different chemical features.

Nevertheless, the apparent scarcity of non-binding examples (n = 1) limits our ability to discern any salient features of a structure-activity relationship.

## Discussion

This study shows that BPA, a molecule which is known to be an endocrine disruptor, displays a weak affinity for amyloid fibers formed by the **P11** peptide, a conserved sequence motif found in the AR-NTD. This interaction seems to be related to specific properties conferred to the oligomerized peptide by the amyloid structure rather than to a binding pocket formed by specific amino-acid side-chains, as indicated by the binding observed between BPA and glutamine homopolymer fibers. The ability of BPA to bind **P11** peptide amyloid fibers is shared by a number of other molecules including some members of the polyphenol family, such as the resveratrol and epigallocatechin gallate. The lack of interaction observed for chloramphenicol suggests that this ability requires specific molecular features that remain to be defined using high-throughput screening. The interaction between BPA and **P11** fibers was evidenced by the WaterLOGSY experiment, an NMR method designed to detect transient binding between a ligand and a large molecular target using water mediated magnetization transfer. In the case of BPA binding to **P11** or polyQ amyloid fibers, this experiment provided us with a strikingly large effect, while other NMR experiments used to evidence transient interactions, such as saturation transfer difference spectroscopy [27] led to undetectable or uninterpretable effects. The large amplitude of the WL signal may therefore be related to specific hydration properties of the amyloid fibers. Indeed, recent studies have revealed that the hydration water dynamics is significantly enhanced after fiber formation of the tau protein [28]. Furthermore, a recent study of the dehydration-related structural deformation of the fungal prion HET-s(218-289) indicates that water can play a significant role in complex amyloid structures as studied by X-ray diffraction [29]. Our results identify the WaterLOGSY experiments as a particularly well adapted method to identify molecules that have an affinity for amyloid fibers, making it a useful tool for the design of future therapies against a number of diseases involving protein misfolding.

The design of ligands able to specifically target the NTD of AR is a subject of intensive research, as this domain is involved in the constitutive and unfaithful induction of the gene expression in the case of castration-resistant prostate cancer [30]. The main difficulty associated with the quest for molecules able to target AR-NTD is related to its intrinsically disordered nature, which prevents the application of conventional approaches based on knowledge of the 3D structure. However, the recent evidence of the ability of an AR-NTD region to reversible form highly ordered structures such as amyloid fibers sheds a new light on this domain [13]. In addition to the conserved **P11** region, AR-NTD contains a number of homopolymer sequences that are potentially involved in the supra-molecular organization of AR, such as a polyQ tract [31]. Furthermore, aggregated forms of AR have been observed in vivo [9], suggesting that the supra-molecular organization of AR into amyloid fibers may have physiological relevance, although any mechanism of action remains to be elucidated. The ability of certain types of molecules to specifically bind to amyloid forms of the receptor may therefore be related to their biological activity. This may be the case for a number of endocrine disruptors such as BPA, whose transient interaction to AR-NTD could increase its local concentration in the vicinity of the receptor and reinforce its activity.

A similar route could be followed to design potent molecules targeting AR. The EPI–001 molecule and its derivatives have been described as potential new drugs promoting the regression of prostate cancer [6, 23]. These molecules share a common molecular framework with BPA. They lack the phenolic protons of BPA, which are replaced by short aliphatic chains. They form covalent adducts with the AR-NTD and block transcriptional activity of AR and its splice variants [23]. The precise mechanism of EPI–001 AR inhibitory activity is still unclear, but our data suggest that its potency may also be related to its ability to bind fibrillar structures of AR, albeit with low affinity.

## Conclusion

Non-specific molecular interactions mediated at the surface of amyloid fibers emerge as novel protein-ligand interactions potentially involved in the mechanisms leading to to amyloid diseases. In the case of androgen receptor, amyloid fibers play an important, albeit enigmatic, role in pathologies such as spinal bulbar muscular atrophy and prostate cancer [9, 11], and should in consequence be considered as potential new therapeutic targets. Our study suggests that the use of NMR WaterLOGSY experiments may be particularly well adapted for the detection of molecular hits aimed at this new family of targets.

## Supporting Information

S1 Spectra Control spectra and WaterLOGSY spectra. This Supporting Information presents various ^1^H NMR spectra from of the different molecules used in this study: regular 1D spectra; WaterLOGSY (WL) spectra in presence of the P11 peptide; control WL spectra in absence of peptide.
(PDF)

S1 NMRData Raw NMR data provided in a zip file. (ZIP)
