# Supplementary material for: NMR WaterLOGSY Reveals Weak Binding of Bisphenol A with Amyloid Fibers of a Conserved 11 Residue Peptide from Androgen Receptor
Source: PLoS One. 2016 Sep 1;11(9):e0161948. doi: 10.1371/journal.pone.0161948 (PMC5008648; doi:10.1371/journal.pone.0161948)
Supplement: S1 Spectra — This Supporting Information presents various 1H NMR spectra from of the different molecules used in this study: regular 1D spectra; WaterLOGSY (WL) spectra in presence of the P11 peptide; control WL spectra in absence of peptide. (PDF) [file pone.0161948.s001.pdf]

Supporting Information
File S1 - Control spectra and WaterLOGSY spectra.

this file is associated to:
NMR WaterLOGSY reveals weak binding of bisphenol A with amyloid fibers of a conserved 11 residue peptide from androgen receptor
Julia Asencio-Hernández, Bruno Kieffer, and Marc-André Delsuc

This Supporting Information presents various ^{1}H NMR spectra from of the different molecules used in this study:

- Regular 1D spectra
- WaterLOGSY (WL) spectra in presence of the P11 peptide
- control WL spectra in absence of peptide

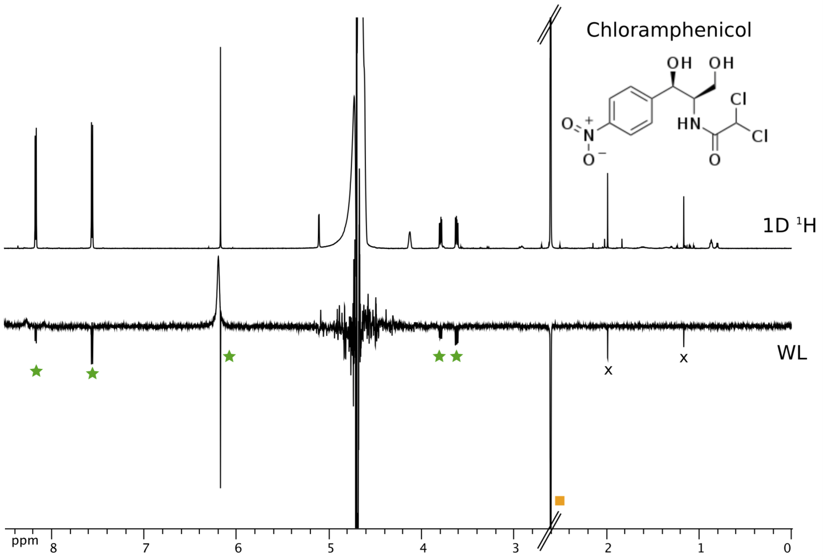

Figure A. WL response of Chloramphenicol in the presence of amyloid fibers formed from peptide P11. Green stars show the Chloramphenicol peaks, orange square indicates DMSO and "×" are impurities. Chloramphenicol is present at 1 mM whereas peptide **P11** is at 200 μM (1/5 dilution of the fibers formed with the peptide **P11** at 1 mM).

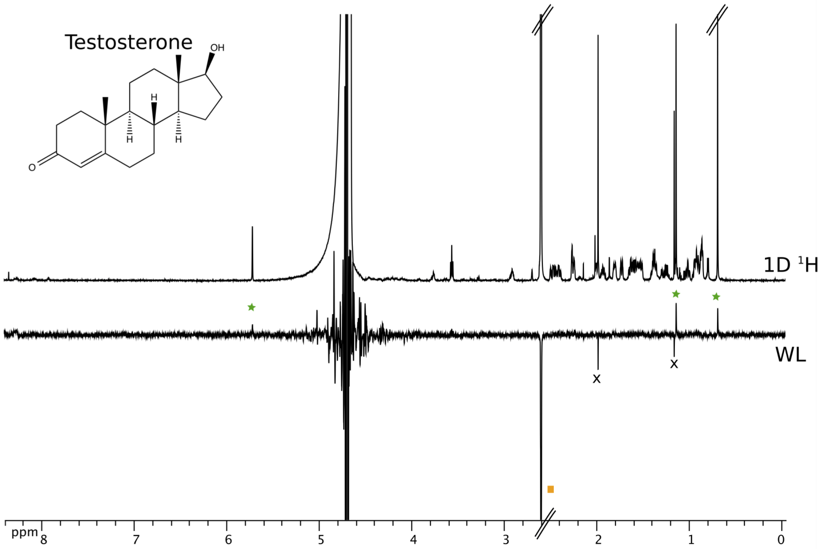

Figure B. WL response of Testosterone in the presence of amyloid fibers formed from peptide P11. Green stars show the Testosterone peaks, orange square indicates DMSO and "×" are impurities. Testosterone is present at 1mM whereas peptide **P11** is at 200 μM (1/5 dilution of the fibers formed with the peptide **P11** at 1 mM).

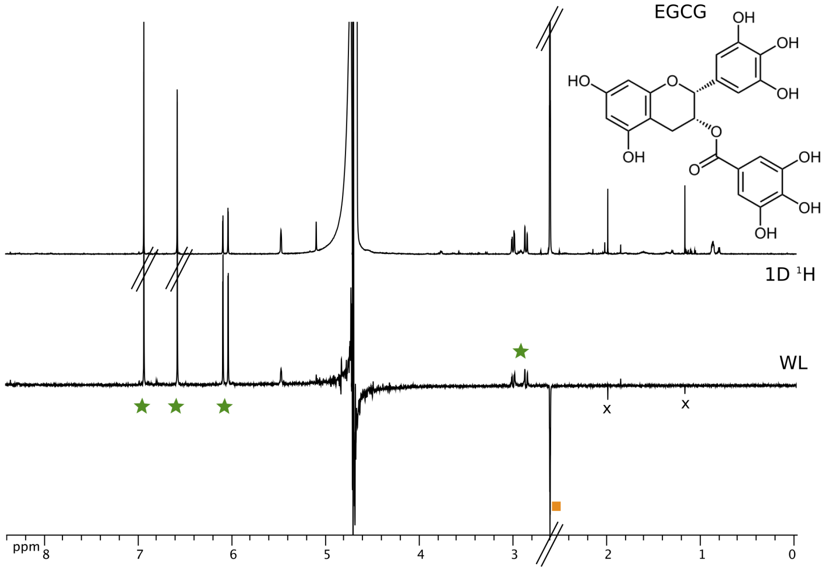

Figure C. WL response of EGCG in the presence of amyloid fibers formed from peptide P11. Green stars show the EGCG peaks, orange square indicates DMSO and “×” are impurities. EGCG is present at 1mM whereas peptide **P11** is at 200 μM (1/5 dilution of the fibers formed with the peptide **P11** at 1 mM).

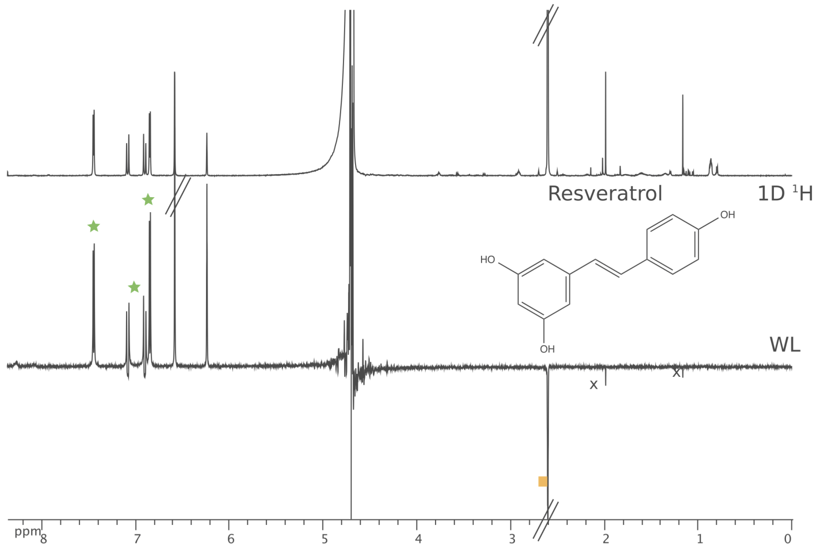

Figure D. WL response of Resveratrol in the presence of amyloid fibers formed from peptide P11. Green stars show the Resveratrol peaks, orange square indicates DMSO and "×" are impurities. Resveratrol is present at 1mM whereas peptide **P11** is at 200 μM (1/5 dilution of the fibers formed with the peptide **P11** at 1 mM).

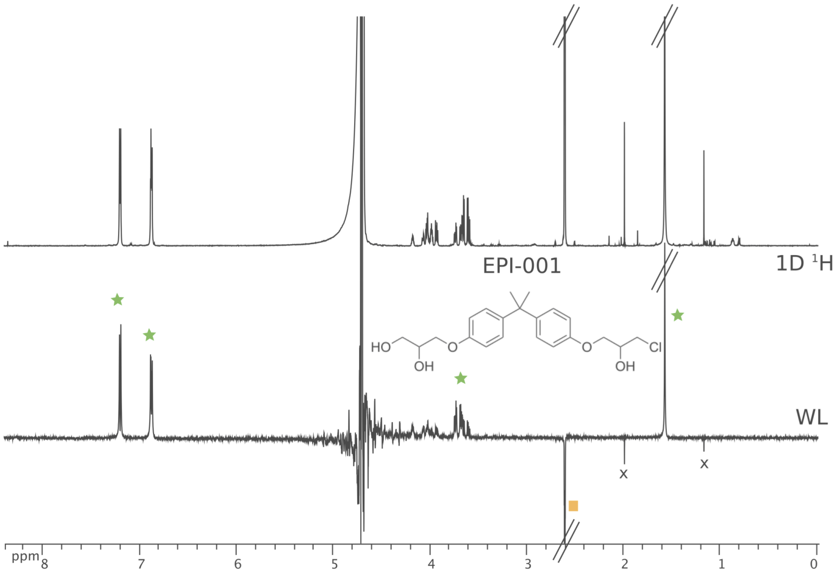

Figure E. WL response of EPI–001 in the presence of amyloid fibers formed from peptide P11. Green stars show the EPI–001 peaks, orange square indicates DMSO and "×" are impurities. EPI–001 is present at 1mM whereas peptide **P11** is at 200 μM (1/5 dilution of the fibers formed with the peptide **P11** at 1 mM).

BPA spectrum with the Cys to Ser mutated peptide **mut**.

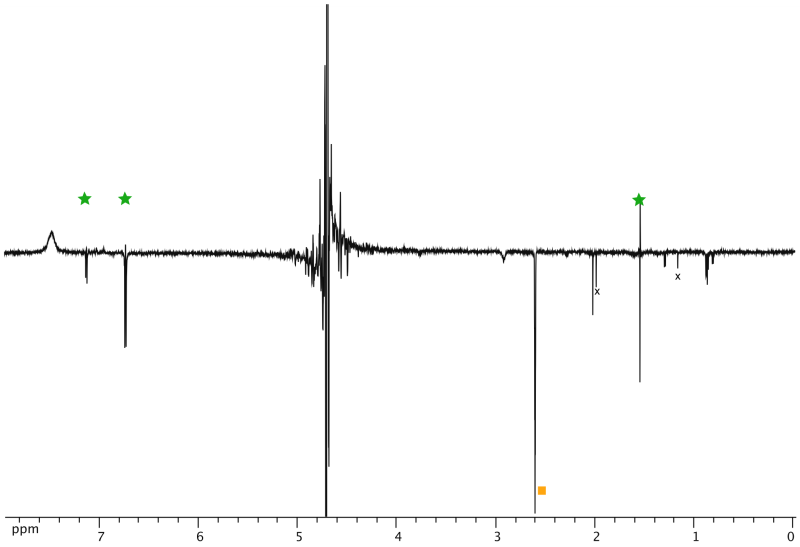

Figure F. WL response of BPA in the presence of peptide mut. Green stars show the BPA peaks and orange square indicates DMSO. BPA and peptide **mut** are present at 1 mM.

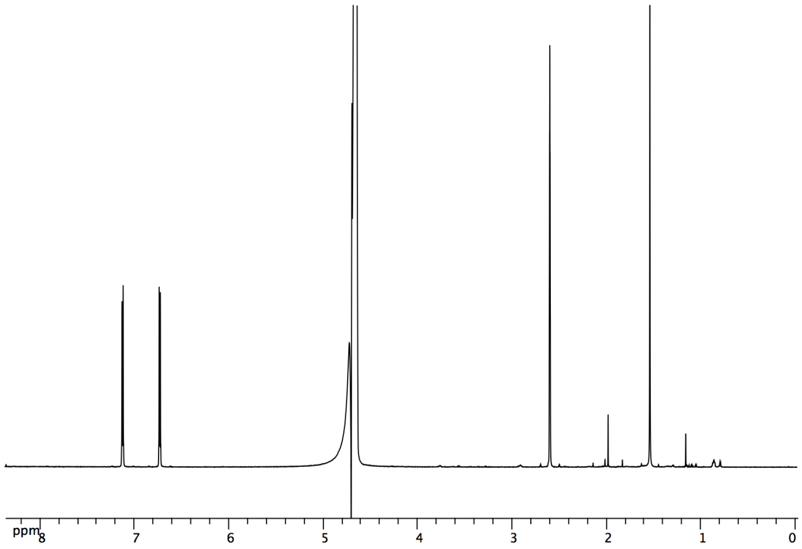

Figure G. 1D ^{1}H of BPA in the presence of amyloid fibers formed from peptide P11.

The following spectra show the control WL experiments for the different molecules alone in solution.

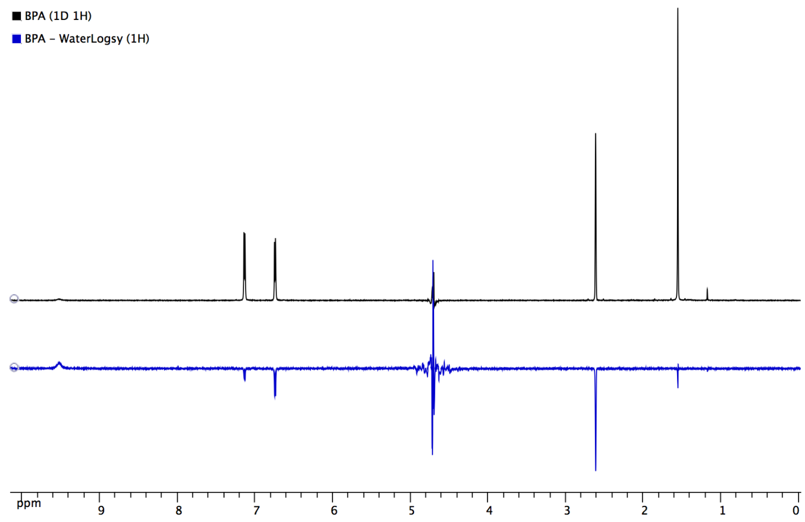

Figure H. Control WL response of BPA alone in solution.

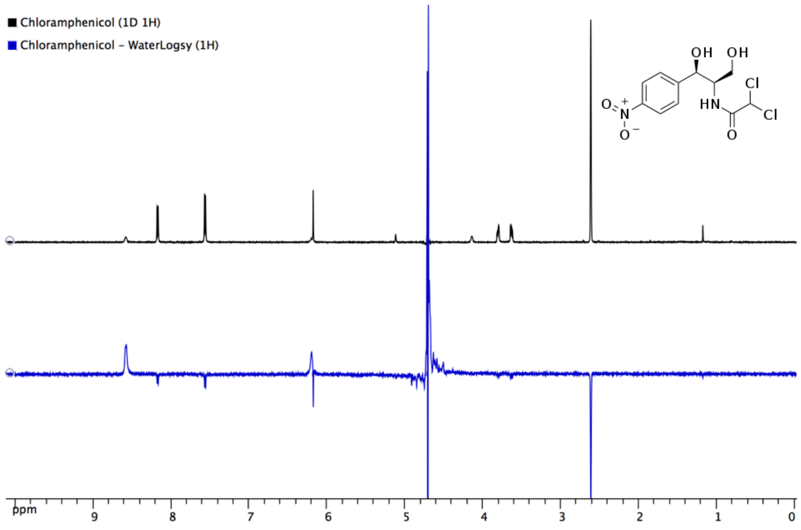

Figure I. Control WL response of Chloramphenicol alone in solution.

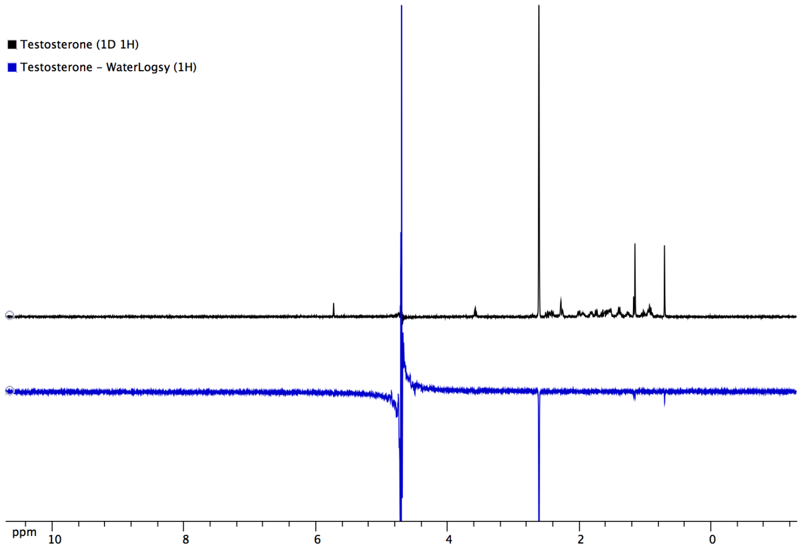

Figure J. Control WL response of Testosterone alone in solution.

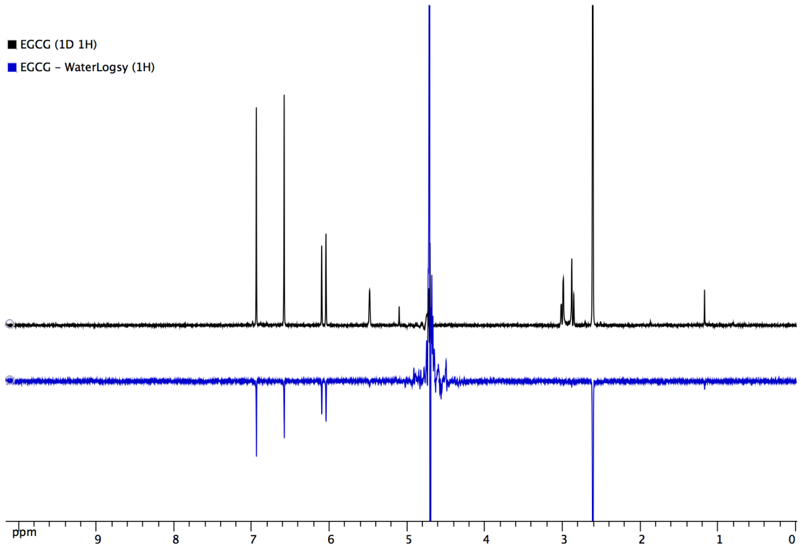

Figure K. Control WL response of EGCG alone in solution.

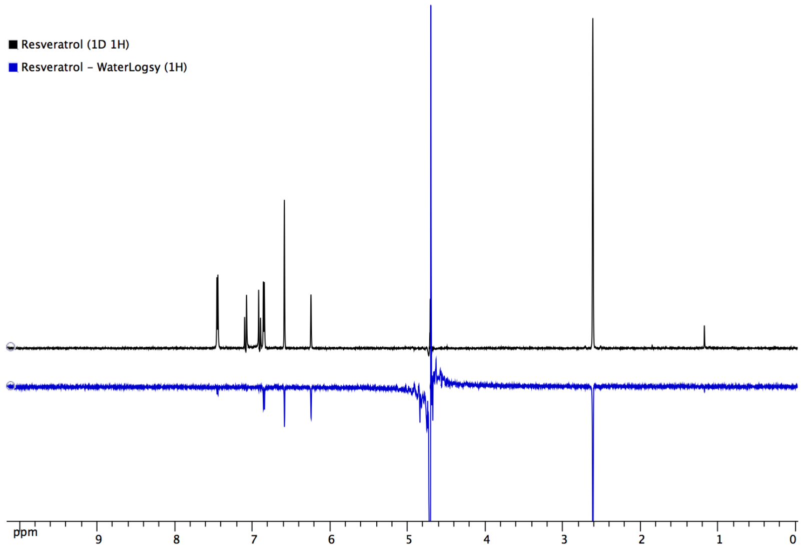

Figure L. Control WL response of Resverastrol alone in solution.

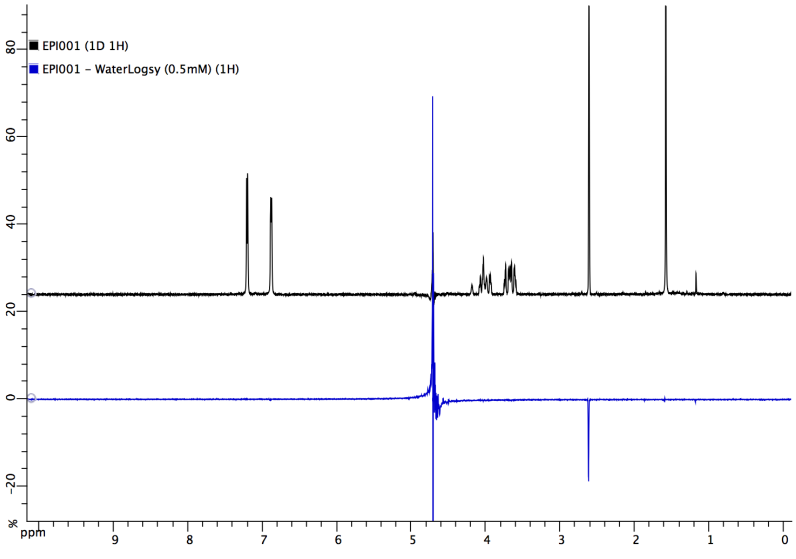

Figure M. Control WL response of EPI–001 alone in solution.

Tabulated values

Values used in Figure 3, 4 & 5

Table A. values used in figure 3

[BPA] μM	80	500	700	900
WL BPA	8.247	51.071	86.99	101.052
WL DMSO	-194.367	-200.979	-200.931	-198.475

Table B. values used in figure 4

[P11] μM	500	200	100	50	20	10	mut	ctrl
WL BPA	203.212	91.972	61.416	47.562	12.172	19.205	-18.679	-2.346
WL DMSO	-203.467	-201.939	-185.213	-207.381	-208.109	-203.84	-200.225	-200.848

Table C. values used in figure 5

	P11	polyQ
WL BPA	61.416	264.338
WL DMSO	-185.213	-195.55
